# Afebrile Pneumonia in a Patient With Multicentric Castleman Disease on Siltuximab: Infection Without Fever on Anti-Interleukin-6 Therapy

**DOI:** 10.7759/cureus.8967

**Published:** 2020-07-02

**Authors:** Philip R Cohen, Mina Nikanjam, Shumei Kato, Aaron M Goodman, Razelle Kurzrock

**Affiliations:** 1 Dermatology, San Diego Family Dermatology, National City, USA; 2 Division of Hematology and Oncology, University of California San Diego, La Jolla, USA; 3 Division of Hematology and Oncology/Division of Blood and Marrow Transplantation, University of California San Diego, La Jolla, USA; 4 Center for Personalized Cancer Therapy, University of California San Diego Moores Cancer Center, La Jolla, USA

**Keywords:** afebrile, castleman, cutaneous, disease, fever, idiopathic, multicentric, siltuximab, tocilizumab, interleukin-6

## Abstract

Castleman disease is a lymphoproliferative disorder characterized by atypical lymph node hyperplasia and systemic symptoms; it can also affect the skin and blood counts. The condition is categorized by the extent of involvement (unicentric or multicentric) and the observed lymph node pathology (hyaline-vascular, plasma cell or mixed cellularity). Pathogenesis also has a role in the classification and treatment of multicentric Castleman disease; this variant can either be related to the presence of human herpesvirus-8 (HHV-8) infection or associated with POEMS (polyneuropathy, organomegaly, endocrinopathy, monoclonal proteins and skin changes) syndrome, or idiopathic. The principal cytokine responsible for causing idiopathic multicentric Castleman disease (IMCD) is interleukin-6 (IL-6). Therefore, treatment with agents that bind to IL-6 (such as siltuximab) or block the IL-6 receptor (such as tocilizumab) has been used. We report a woman with IMCD who was successfully being treated with siltuximab; her cutaneous manifestations and systemic disease (lung and lymph nodes) improved within three months. However, nine months after starting siltuximab, she developed a worsening cough and new infiltrates in the right lung on positron emission tomography/computed tomography (PET/CT) scan; there were no other constitutional symptoms such as fever, night sweats or fatigue. Differential diagnosis included Castleman disease recurrence, lung neoplasm and infection. Her pulmonary symptoms and infiltrates on scan resolved after treatment with systemic levofloxacin, indicating that she had an antibiotic-sensitive afebrile pneumonia. We postulate that her siltuximab therapy blocked the IL-6-associated fever and constitutional symptoms that normally are a hallmark of pneumonia. Therefore, patients who are receiving medications such as siltuximab and tocilizumab that block the IL-6 pathway and impair the acute phase inflammatory response may fail to manifest constitutional symptoms such as fever when infected.

## Introduction

Castleman disease, a lymphoproliferative disorder, can affect not only lymph nodes but also extranodal sites. It is categorized not only by the extent of involvement (unicentric or multicentric), but also by pathology (hyaline-vascular, plasma cell or mixed cellularity). Multicentric Castleman disease is also classified by pathogenesis: idiopathic or human herpesvirus-8 (HHV-8)-related or POEMS (polyneuropathy, organomegaly, endocrinopathy, monoclonal proteins and skin changes) syndrome-associated [1-4]. Castleman disease appears to be more common in Asians, especially Japanese, for reasons that are unclear.

Siltuximab is a chimeric (human-murine) anti-interleukin-6 (IL-6) monoclonal antibody. It has a high affinity for binding with human IL-6. It is a novel, targeted therapy for the treatment of patients with idiopathic multifocal Castleman disease (IMCD) and is approved by the Food and Drug Administration (FDA) [5-8].

A woman with cutaneous and systemic manifestations of HHV-8-negative IMCD was successfully treated with siltuximab. She was maintained on therapy after experiencing resolution of her disease-related skin lesions and other manifestations. However, she developed cough and radiologically confirmed pneumonia without any fever or other constitutional symptoms; her pulmonary infection cleared with systemic antibiotics. We hypothesize that her siltuximab therapy blocked the IL-6-associated fever and constitutional symptoms that patients with pneumonia typically develop; therefore, clinicians need to be aware that systemic infections may not present in their usual clinical manner in patients who are receiving a targeted therapy that interferes with the action of IL-6 (such as siltuximab) or the IL-6 receptor (such as tocilizumab).

## Case presentation

A 60-year-old Asian woman on siltuximab (11 mg/kg) infusion every three weeks for biopsy-proven cutaneous and systemic IMCD (plasma cell type) presented with recent onset of cough. However, she was afebrile and had no other constitutional symptoms.

Her disease onset began 14 years earlier. She presented with skin lesions as well as a persistent cough and hemoptysis. A CT scan only revealed mild bronchiectasis of her right upper lobe and right lower lobe of her lung with bronchial wall thickening; in addition, heterogeneous areas of nodular and linear interstitial thickening with ground-glass opacification were observed in the right upper lobe and the right middle lobe. Her bronchoscopy was normal, and the bronchoalveolar lavage was negative for bacteria, fungi and mycobacteria.

Her condition remained undiagnosed for another 11 years. Biopsies of her skin lesions from the back and groin then showed a polytypical plasma cell infiltrate with B-cell hyperplasia. The kappa to lambda staining ratio was normal (3:1), and light chain restriction was not demonstrated. The pathological diagnosis was consistent with Castleman disease, plasma cell variant. HHV-8 was negative and she had no diagnostic features of POEMS syndrome.

**Figure 1:**
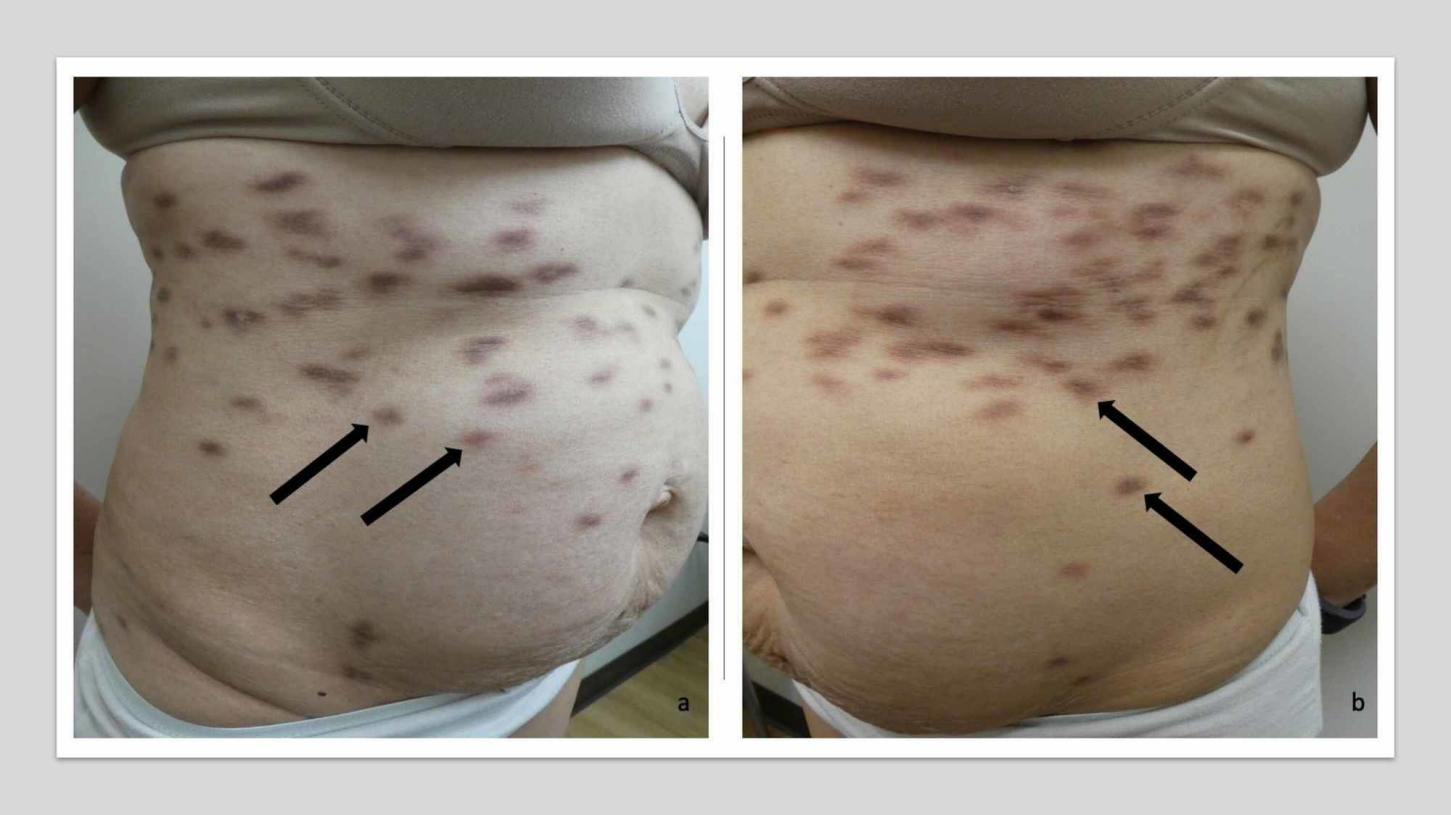
Cutaneous Castleman disease lesions in an Asian woman with idiopathic multicentric Castleman disease (IMCD) Dark brown patches and plaques of cutaneous Castleman disease (black arrows) on the right flank (a) and left flank (b) of an Asian woman with IMCD. The lesions resolved after she began treatment with siltuximab.

She was then referred to our institution and we planned to start siltuximab. At that time, she had a cough, mild hemoptysis, and numerous hyperpigmented brown patches and plaques on her lower chest and abdomen, bilateral flanks, back and buttocks, axillae and groin, and proximal arms and legs (Figure 1). She told us that new skin lesions appeared every couple of weeks.

**Figure 2:**
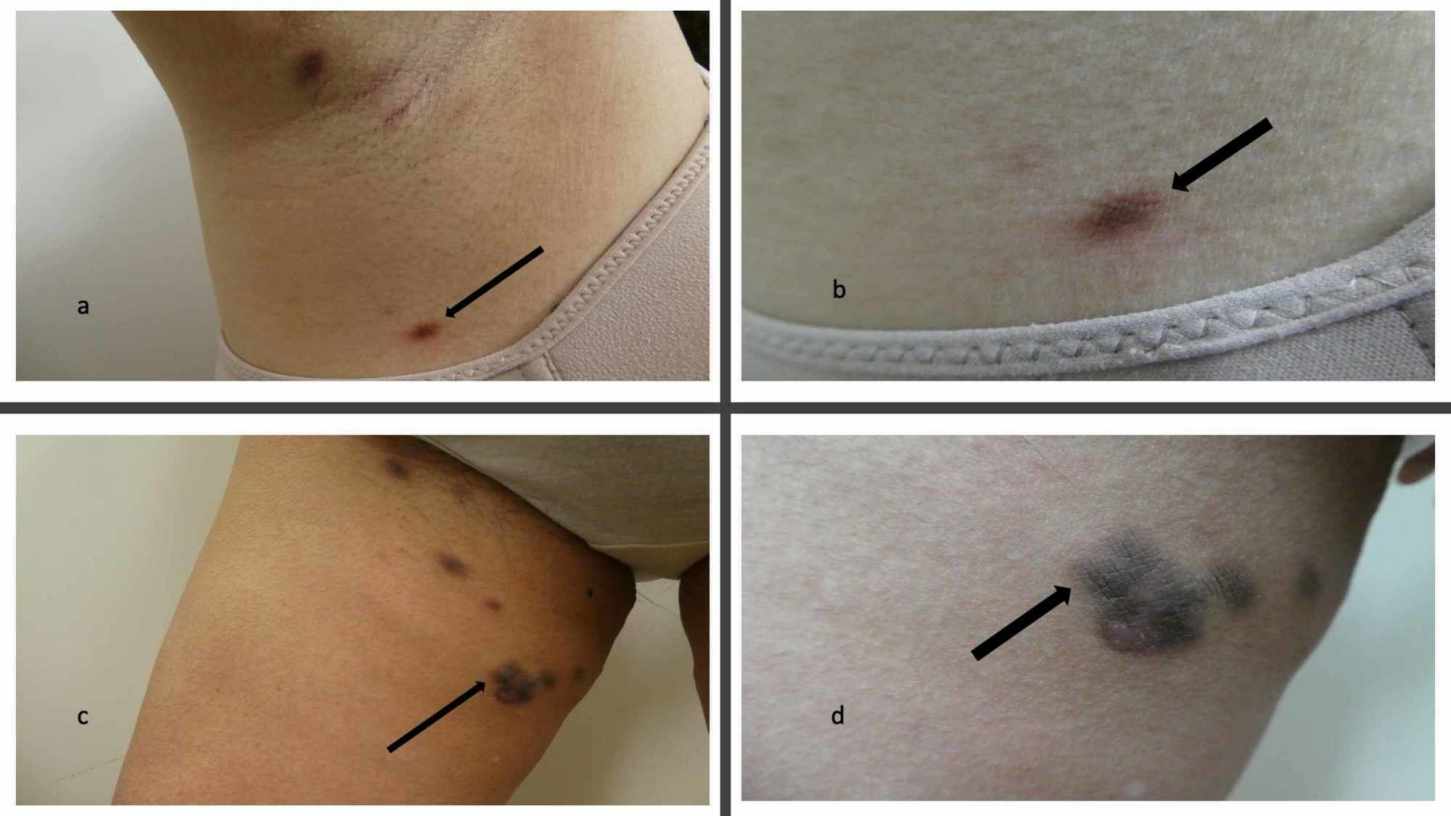
Skin biopsy sites of cutaneous Castleman disease in an Asian woman with idiopathic multicentric Castleman disease (IMCD) Hyperpigmented brown patches and plaques of cutaneous Castleman disease on the right axilla (a and b) and right thigh (c and d) of an Asian woman with IMCD. Distant (a and c) and closer (b and d) views of the skin biopsy sites (black arrows) which confirmed the cutaneous involvement of her IMCD.

Biopsies were performed from plaques on her right axilla and right thigh (Figure 2). They were again consistent with Castleman disease, showing a cutaneous B-cell infiltrate with germinal center formation and polytypic plasma cells; the kappa to lambda evaluation also revealed polytypic plasma cells. Staining for HHV-8 was again negative.

A baseline positron emission tomography/computed tomography (PET/CT) scan showed mildly enlarged lymph nodes in the neck, chest and abdomen with mildly elevated fluorodeoxyglucose activity. A right submental lymph node biopsy also showed Castleman disease. Pertinent laboratory study results included an elevated erythrocyte sedimentation rate, anemia (hemoglobin = 11.5 g/dL) and thrombocytosis (platelets = 578,000/µL). 

She was started on siltuximab at a dose schedule of 11 mg/kg intravenously every three weeks. Within three months, her skin lesions had all flattened and several began to fade. No new lesions appeared. Her cough and hemoptysis resolved. Follow-up PET/CT scans after three and six months showed improvement in imaging abnormalities.

**Figure 3:**
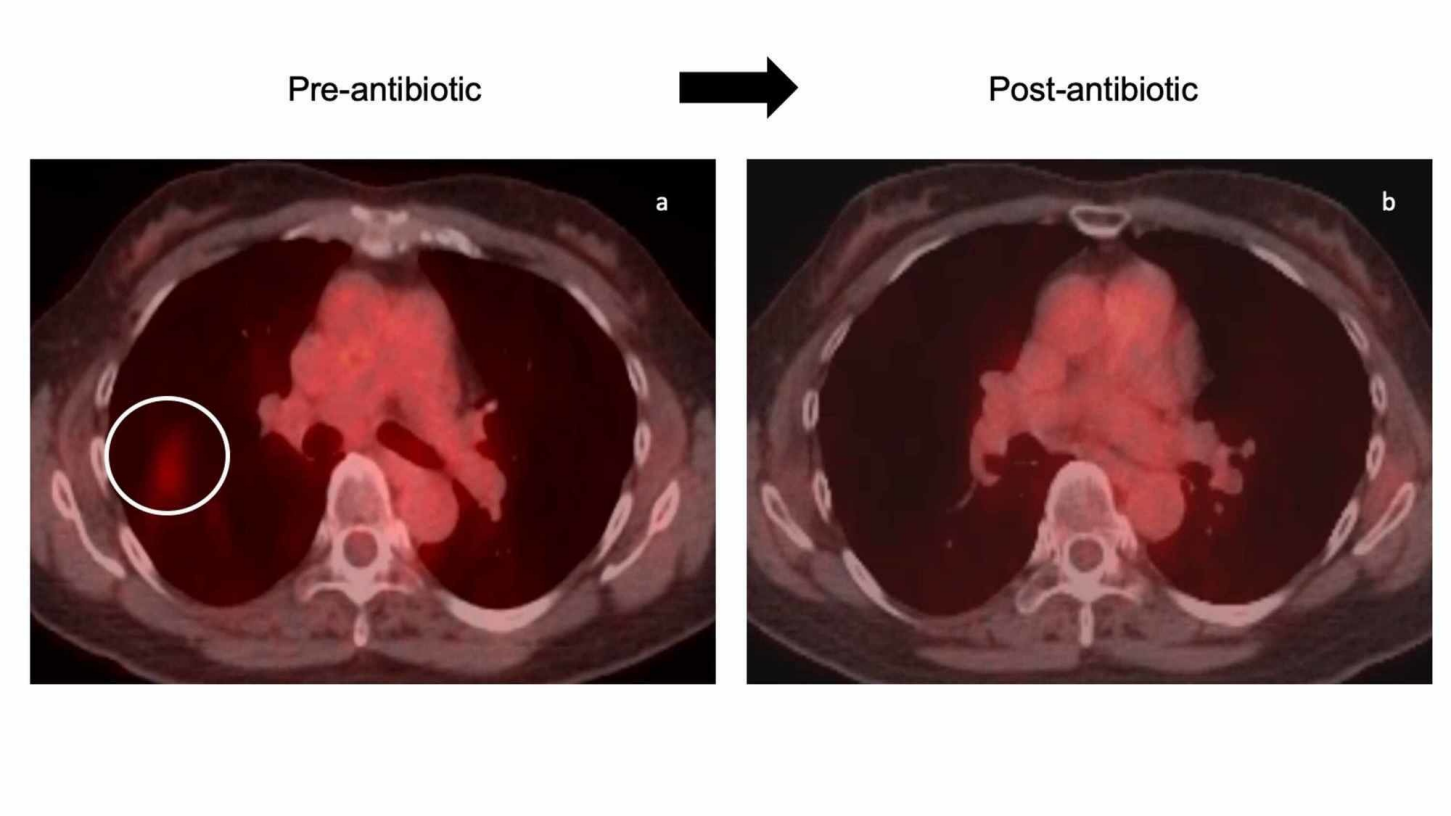
Positron emission tomography/computed tomography (PET/CT) scans demonstrating evidence of active infection and resolution of an afebrile pneumonia An Asian woman with cutaneous and systemic idiopathic multicentric Castleman disease (IMCD) who had been on siltuximab (11 mg/kg intravenously every three weeks) for nine months developed a cough and new patchy infiltrates in the right lung (red area within the white circle) on her staging PET/CT scan (a). Her cough resolved after seven days of systemic antibiotics (oral levofloxacin 750 mg daily). Follow-up staging PET/CT scan (b) three months later showed that the patchy infiltrates had disappeared.

After nine months of treatment, however, her cough recurred. She had no fever and no other constitutional symptoms such as arthralgias, myalgias, fatigue, night sweats or weight loss. Her skin lesions were continuing to diminish. Her staging PET/CT scan showed new patchy infiltrates in the right lung (Figure 3).

The principal differential diagnoses of her pulmonary symptom (cough) and radiographic findings included a recurrence or progression of her Castleman disease, a new pulmonary tumor and an afebrile pneumonia. An infectious etiology was favored, and she was empirically treated with levofloxacin 750 mg daily for seven days.

All of her pulmonary symptoms had resolved when she was seen at her follow-up visit two weeks after starting oral antibiotics. Her staging PET/CT scan three months later showed disappearance the patchy infiltrates that had been observed on the prior PET/CT scan. Correlation of the clinical features and radiographic studies established the diagnosis of an afebrile pneumonia without any associated constitutional symptoms.

She has been maintained on her siltuximab every three weeks. Her IMCD continues to improve. She has not had another episode of cough.

## Discussion

The management of Castleman disease is based upon the specific subtype. Unicentric Castleman disease, with a solitary focus of lymphadenopathy, may be successfully treated by excision of the affected overgrowth of lymphoid tissue. In unresectable or recurrent disease, other modalities such as radiotherapy, systemic corticosteroids and rituximab might be warranted [1,2,4].

For HHV-8-positive multicentric Castleman disease patients, rituximab can be effective. Antiretroviral therapy should also be included for HIV-positive patients. Siltuximab is not recommended since the agent does not bind to viral IL-6 [1,2,4].

POEMS syndrome-associated multicentric Castleman disease treatment needs to be directed towards the monoclonal plasma cell proliferation. There is a report of successful treatment of a woman with multicentric Castleman disease who had several features of POEMS syndrome with anakinra, an interleukin-1 (IL-1) receptor antagonist [9]. 

In the case of IMCD, the B-cell proliferation and laboratory abnormalities are driven by elevated levels of IL-6 [3,8]. Therefore, novel treatments focused at the etiology for the pathogenesis of this variant of Castleman disease are used. Currently, the initial management for IMCD usually includes either siltuximab (approved for Castleman disease in the United States of America) or tocilizumab (approved in Japan) [3,10,11].

Siltuximab binds IL-6 and prevents it from binding to its receptor. Tocilizumab is a humanized IL-6 receptor antagonist that blocks the IL-6 receptor. Tocilizumab is approved in the United States of America for rheumatoid arthritis (and as mentioned, in Japan, for Castleman disease) [3,10,11]. 

Cutaneous involvement of multicentric Castleman disease is uncommon [12]. It is almost always associated with the plasma cell type of IMCD [12-17]. Rarely, patients with the hyaline-vascular type of cutaneous multicentric Castleman disease have been reported [18].

Cutaneous manifestations of IMCD are also typically associated with systemic involvement; however, Castleman disease limited only to the skin has been observed [17]. Individual patients with cutaneous involvement of multicentric Castleman disease have had successful clearing of their skin lesion when treated with systemic corticosteroids, single agent chemotherapy (cyclophosphamide), combination chemotherapy such as eight cycles of CHOP (cyclophosphamide, adriamycin, vincristine and prednisone) or thalidomide [12,13,16]. Similar to our patient, partial or complete resolution of cutaneous manifestations of Castleman disease has been achieved with siltuximab treatment [14,15,17].

IL-6 is responsible for causing inflammatory symptoms (such as fever, night sweats, weight loss and fatigue) in Castleman disease patients [4,8,19]. Infections, such as pneumonia, usually cause similar constitutional symptoms; although the etiology of these symptoms may be multifactorial, it is reasonable to attribute them, at least in part, to IL-6. Hence, a patient who is receiving an agent that blocks IL-6 and thereby diminishes or eliminates the inflammatory response from this endogenous cytokine might not develop fever or night sweats or fatigue related to a systemic infection [4,20].

Our patient was regularly receiving siltuximab, which binds her IL-6 and interferes with its normal function. The only presenting symptom of her pneumonia was cough; she was afebrile and had no constitutional symptoms. However, new patchy infiltrates had appeared on her staging PET/CT scan. In addition to pneumonia, these findings in an IMCD patient with a history of lung involvement could represent recurrent or progressive Castleman disease or a pulmonary tumor.

Her prompt response to an oral antibiotic (levofloxacin) and clearing of the radiographic findings on the subsequent PET/CT scan confirmed our suspected diagnosis of infection. Had her cough persisted or the PET/CT scan remained unchanged, additional bronchoscopy evaluation for biopsy and cultures would have been necessary. It is important for physicians who manage patients on medications such as siltuximab and tocilizumab, which diminish or eliminate their innate IL-6 signaling, to realize that these individuals may not be able to mount an acute phase response; this might not only increase their risk of infections, but also result in an absence of infection-associated constitutional symptoms such as fever, night sweats and fatigue.

## Conclusions

IMCD is a B-cell lymphoproliferative disorder that is characterized by hyperplasia of lymph nodes in addition to systemic symptoms and findings including, albeit rarely, cutaneous manifestations. IL-6 has been identified as the principal cytokine responsible for causing Castleman disease and attendant constitutional symptoms; therefore agents that bind to IL-6 (such as siltuximab) or block the IL-6 receptor (such as tocilizumab) have been used to successfully manage IMCD patients. We observed a woman with cutaneous and systemic IMCD whose disease manifestations were successfully treated with siltuximab; however, she developed a PET/CT scan-confirmed pneumonia that presented only with cough and no other constitutional symptoms such as fever, night sweats, fatigue, arthralgias, myalgias or weight loss; her cough and changes noted on PET/CT scan promptly resolved after treatment with systemic antibiotics. It is important for clinicians to be aware that patients who are receiving medications such as siltuximab and tocilizumab, which would block the IL-6 pathway, not only may have an increased risk of acquiring infection (since their acute phase inflammatory response is impaired) but also can have serious infections that clinically present without constitutional symptoms such as fever.
